# Tangeretin Suppresses LUAD via SSTR4 Downregulation: Integrated Bioinformatics and Functional Validation

**DOI:** 10.3390/ijms27021074

**Published:** 2026-01-21

**Authors:** Yizhen Yuan, Yongfu Wang, Wei Liu, Changmin Liu, Yajing Xue, Pengzhuo Tao, Shilin Chen, Chi Song

**Affiliations:** 1 School of Pharmacy, Chengdu University of Traditional Chinese Medicine, Chengdu 611137, China; yuanyizhen2025@163.com (Y.Y.); wyf@stu.cdutcm.edu.cn (Y.W.); 18617077221@163.com (W.L.); liuchangmin0908@163.com (C.L.); xueyajing1432@163.com (Y.X.); pengzhuotao998@163.com (P.T.); 2 Institute of Herbgenomics, Chengdu University of Traditional Chinese Medicine, Chengdu 611137, China; 3 Hubei Shizhen Laboratory, Wuhan 430061, China

**Keywords:** SSTR4, LUAD, tangeretin, A549, RNA-seq

## Abstract

Lung adenocarcinoma (LUAD) remains the leading cause of cancer-related mortality worldwide, highlighting the urgent need for novel therapeutic targets. While the role of the somatostatin receptor (SSTR) family is well established in neuroendocrine tumors, their expression patterns, clinical significance, and therapeutic potential in LUAD are not fully understood. In this study, comprehensive analyses of publicly available databases, including TCGA, GSCA, and TIMER, revealed that SSTR4 transcriptional expression is significantly downregulated in LUAD tissues compared to adjacent normal lung tissues. Moreover, low SSTR4 expression correlates with advanced tumor stage, remodeling of the immune microenvironment, and decreased overall survival in patients with LUAD. Using the PRESTO-Tango system, we identified tangeretin (TAN) as a potential ligand for SSTR4. Functional assays demonstrated that SSTR4 knockdown markedly enhanced TAN-mediated proliferative, migratory, and survival inhibitory effects in LUAD cells. Subsequent RNA sequencing and pathway enrichment analyses revealed that the loss of SSTR4 altered the effects of TAN from extracellular matrix remodeling to disruption of calcium homeostasis and energy metabolism disorders, elucidating the mechanism underlying the enhanced antitumor activity. Collectively, these findings establish SSTR4 as a critical tumor suppressor and prognostic biomarker in LUAD and highlight the therapeutic potential of targeting the TAN–SSTR4 signaling axis. These results provide novel insights into the biological functions of SSTR family members in LUAD.

## 1. Introduction

Lung cancer is the leading cause of cancer-related deaths worldwide. LUAD accounts for around 40% of all lung cancer cases [1,2]. Over the past decade, the incidence of LUAD has continued to rise not only among long-term smokers, but also predominantly affects women and non-smokers [3]. Despite advances in treatment, including immune checkpoint inhibitors (ICBs) targeting PD-1/PD-L1 [4,5] and targeted therapies for driver mutations such as EGFR and KRAS [6,7], LUAD remains a significant clinical and public health challenge. The dismal five-year overall survival rate of 5–20% is a consequence of several intractable challenges, including late-stage detection, profound metastatic propensity, pervasive therapeutic resistance, and a lack of curative systemic modalities [8,9]. Furthermore, resistance can develop even after prolonged use of these immunotherapies [10]. Therefore, elucidating the molecular mechanisms underlying LUAD initiation, progression, metastasis, and resistance, as well as identifying novel biomarkers and therapeutic targets, is crucial for improving diagnostic and therapeutic strategies and enhancing patient survival.

Tangeretin (TAN), a polymethoxyflavone (PMF) predominantly found in the peel of citrus fruits, exhibits favorable lipophilicity and high oral bioavailability owing to its fully methoxylated molecular scaffold [11]. Notably, its toxicity profile is considerably more favorable compared to that of most conventional chemotherapeutic agents [12]. Since TAN was discovered, extensive research has shown that TAN modulates critical signaling pathways—such as MAOA/NF-κB, NF-κB/ICAM-1, AMPK, and Wnt/β-catenin—across various cancer models, including those of lung, liver, breast, and colorectal cancers [13,14,15,16], thereby suppressing tumor cell proliferation and invasion and reversing epithelial-mesenchymal transition. Other PMFs, such as nobiletin and 5-methoxyflavone, have been shown in LUAD models to induce cell cycle arrest and inhibit proliferation by downregulating the PI3K-AKT-mTOR or PI3K-AKT-GSK3β-β-catenin-Cyclin D1 signaling axes [17,18]. However, the precise role of TAN in LUAD and its underlying molecular targets remains to be systematically characterized.

The somatostatin receptor (SSTR) family comprises five G-protein-coupled receptor (GPCR) subtypes, designated as SSTR1 to SSTR5, which are encoded by distinct genes located on different chromosomes. These receptors have a typical seven-transmembrane structure [19,20] and are widely distributed in hepatic, gastrointestinal, pulmonary, and endocrine tissues [21]. By binding to their endogenous ligand, somatostatin (SST), SSTRs regulate multiple signaling pathways, including adenylate cyclase activity, ion channel function, and intracellular calcium dynamics [22]. This inhibits hormone secretion, cell proliferation, and differentiation processes. Under pathological conditions, SSTRs exhibit significantly elevated expression in neuroendocrine tumors (such as small cell lung cancer and pulmonary carcinoid tumors) and LUAD [23,24]. SSTR2 expression is the most prevalent among these, followed by SSTR3, SSTR5, and SSTR1, while SSTR4 expression is rare. Abnormal activation of SSTR4 influences cytoskeletal reorganization and Rho GTPase activity by regulating pathways such as MAPK and PI3K/AKT/mTOR [25], which are closely correlated with tumor growth, invasion, and poor prognosis.

In recent years, significant clinical progress has been achieved in the development of drugs targeting SSTRs. Radioisotope-labeled somatostatin analogues such as ^177^Lu-DOTATATE (Lutathera^®^) have been approved for treating gastroenteropancreatic neuroendocrine tumors [26], enabling precision radiotherapy by targeting SSTR2. Long-acting formulations such as octreotide and Somatuline are widely used to control symptoms and suppress growth in acromegaly and neuroendocrine tumors [27]. The novel, multi-receptor targeted drug pasireotide (Signifor^®^) exhibits a high affinity for SSTR1, 2, 3, and 5 and is used to treat Cushing’s disease [28]. The development of these drugs highlights the significant value of SSTRs as diagnostic and therapeutic targets and provides new directions for precision cancer therapy. In addition, according to Patsnap Synapse statistics (https://synapse.patsnap.com/ (accessed on 9 October 2025)), multiple drugs targeting SSTRs or different SSTR subtypes have been approved for market release or are currently in clinical research phases. Radiopharmaceuticals and diagnostic agents targeting this receptor are attracting significant attention in particular [29,30].

SSTR4 is physiologically expressed at high levels in the placenta, lung, and enteric neuronal tissue. Immunohistochemical and single-cell sequencing analyses have demonstrated that SSTR4 co-localizes with CD163^+^ M2-type tumor-associated macrophages in colorectal cancer—particularly the microsatellite-instable subtype—as well as in triple-negative breast cancer and melanoma [31,32]. In vitro studies have demonstrated that SSTR4 activation suppresses tumor cell proliferation via the Gi/o–cAMP pathway and concurrently attenuates CD8^+^ T-cell infiltration through β-arrestin-2–mediated signaling [33,34]. Thus, SSTR4 exerts a dual role—directly restraining tumor growth while fostering an immunosuppressive milieu—and its ligands are already under investigation for radionuclide imaging and pain control. Nevertheless, the expression profile and functional significance of this receptor in LUAD remain to be systematically elucidated.

In this study, we first conducted a systematic evaluation of the expression profiles and prognostic value of the SSTR family in LUAD based on multiple public databases. In consideration of the preceding bioinformatics results, it was determined that SSTR4 in LUAD exhibits substantial downregulation, which is indicative of a poor prognosis. Furthermore, a positive correlation was identified between SSTR4 and CD8+ T cells, as well as M1-type macrophages, among other characteristics that are deemed to be amenable to therapeutic intervention. Consequently, it is hypothesized that this may emerge as a prospective therapeutic target. A preliminary investigation was conducted on the initial data obtained by the research team using the self-developed high-throughput PRESTO-Salsa and CRIS-PRa/i platforms. The findings indicated a strong probability that TAN is a potential ligand for SSTR4. To validate its function, we employed PRESTO-Tango technology to confirm that TAN activates SSTR4 and induces β-arrestin recruitment, indicating this ligand modulates SSTR4 signaling via a β-arrestin-dependent pathway. Building on this, we further assessed the effects of the TAN-SSTR4 axis—on A549 cell proliferation, migration, and apoptosis—and employed transcriptome sequencing to explore its potential signaling pathways. Our findings reveal that SSTR4 mediates the antitumor effects of TAN in LUAD, thereby providing a theoretical basis and experimental support for promoting this receptor into novel targeted therapies for LUAD. 

## 2. Results

### 2.1. Differential Analysis of Somatostatin Receptor Family Expression in LUAD

**Figure 1:**
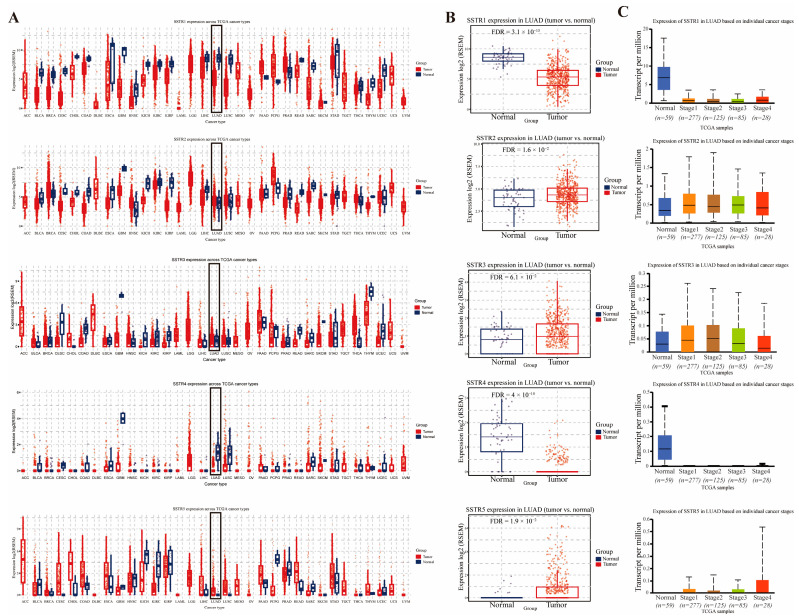
Expression profiles of somatostatin receptor (SSTR) family in LUAD tissues and normal tissues. (**A**) The expression levels of the SSTR family in different tumor types in the TCGA database were determined by GSCA. (**B**) Comparison of differential expression of SSTR1, SSTR2, SSTR3, SSTR4, and SSTR5 in LUAD. The expression of all genes in tumor tissues was significantly different from that in normal tissues. (**C**) Expression of SSTR1 to SSTR5 in LUAD, based on samples from the TCGA database, grouped by clinical stage (from stage I to IV). A box-and-line plot was used to show the distribution of gene expression in different stages. The vertical axis indicates the expression per million transcripts, and the horizontal axis indicates the different clinical stages. The median, upper and lower quartiles, and possible outliers in the box-and-line plots provide information on the centralized trends and variability of gene expression in each stage.

To investigate expression differences of the SSTR family across various cancer types, we employed GSCA to analyze the mRNA levels of the SSTR family. Research findings indicate that there are significant differences in the expression levels of members of the SSTR family across various cancer tissues, including Glioblastoma Multiforme (GBM), Low-Grade Glioma (LGG), Testicular Germ Cell Tumors (TGCT), and LUAD. Specifically, SSTR2, SSTR3, and SSTR5 expression levels were significantly higher in LUAD tissue than in normal tissue, while SSTR1 and SSTR4 expression levels were significantly lower (Figure 1A). Concurrent analysis of SSTR-family expression in LUAD revealed a divergent pattern: SSTR1 and SSTR4 were markedly reduced relative to normal lung, with SSTR4 showing the steepest decline, whereas SSTR2, SSTR3, and SSTR5 were significantly upregulated, among which SSTR5 displayed the most pronounced increase (Figure 1B). Furthermore, SSTR family members exhibited significant expression differences across LUAD patients with varying clinical stages (Figure 1C). As cancer progressed, SSTR5 expression levels increased in patients with later stages. In contrast, SSTR1 and SSTR4 expression decreased, potentially indicating their association with tumor progression.

### 2.2. Survival Analysis of the Somatostatin Receptor Family in LUAD

**Figure 2:**
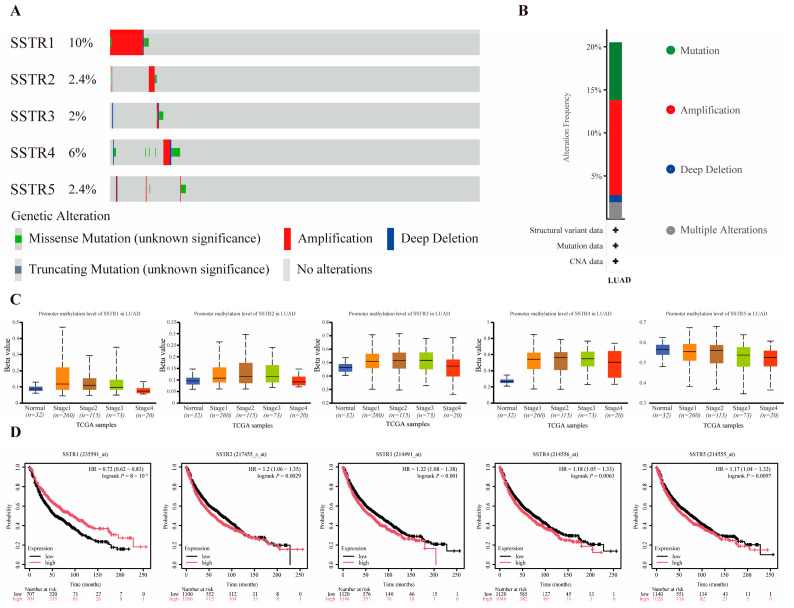
Association Analysis of Mutations, Methylation, and Prognosis in the SSTR Family in LUAD. (**A**,**B**) Mutation profile of SSTR family genes in LUAD. Red indicates gene amplification, blue indicates deep deletion, green indicates missense mutation, gray indicates truncation mutation, and light gray indicates no genetic variation detected. (**C**) Promoter methylation levels of SSTR family genes in LUAD. Box-and-whisker plots show the distribution of gene expression at different methylation levels (from normal to hypermethylated). The black dashed line indicates the median expression of all samples. (**D**) Demonstrates the impact of SSTR1 to SSTR5 expression levels in LUAD on overall patient survival. Survival probabilities for the high-expression group (red) and low-expression group (black) were compared using Kaplan–Meier survival curves. The vertical axis indicates the survival probability, and the horizontal axis indicates the follow-up time (months). The risk ratio (HR) for SSTR family members, their 95% confidence intervals, and the *p*-value of the log-rank test are provided in the figure, and these statistics were used to assess the statistical significance of the differences in patient survival between groups with different expression levels.

SSTR1–SSTR5 exhibited a low-frequency alteration landscape in our cohort, with mutation rates ranging from 4% to 16% and predominantly comprising variants of unknown significance (missense and truncating); copy-number analysis revealed sporadic amplifications and deep deletions, all occurring at frequencies <20% (Figure 2A). Among these events, amplification was the most prevalent alteration type across the SSTR family (Figure 2B). Promoter methylation analysis revealed significant stage-dependent increases in methylation levels for SSTR2, SSTR3, and SSTR4 (Figure 2C). Previous research by our team revealed markedly reduced SSTR4 expression levels compared to normal tissues, potentially due to its silencing by high promoter methylation. Furthermore, survival analysis showed that the expression of SSTR family members is closely associated with prognosis in LUAD patients. High expression of SSTR1 is associated with a favorable prognosis, whereas elevated expression of SSTR2, SSTR3, SSTR4, and SSTR5 predicts lower overall survival rates (Figure 2D).

### 2.3. Correlation Between Expression and Immune Infiltrates in LUAD

**Figure 3:**
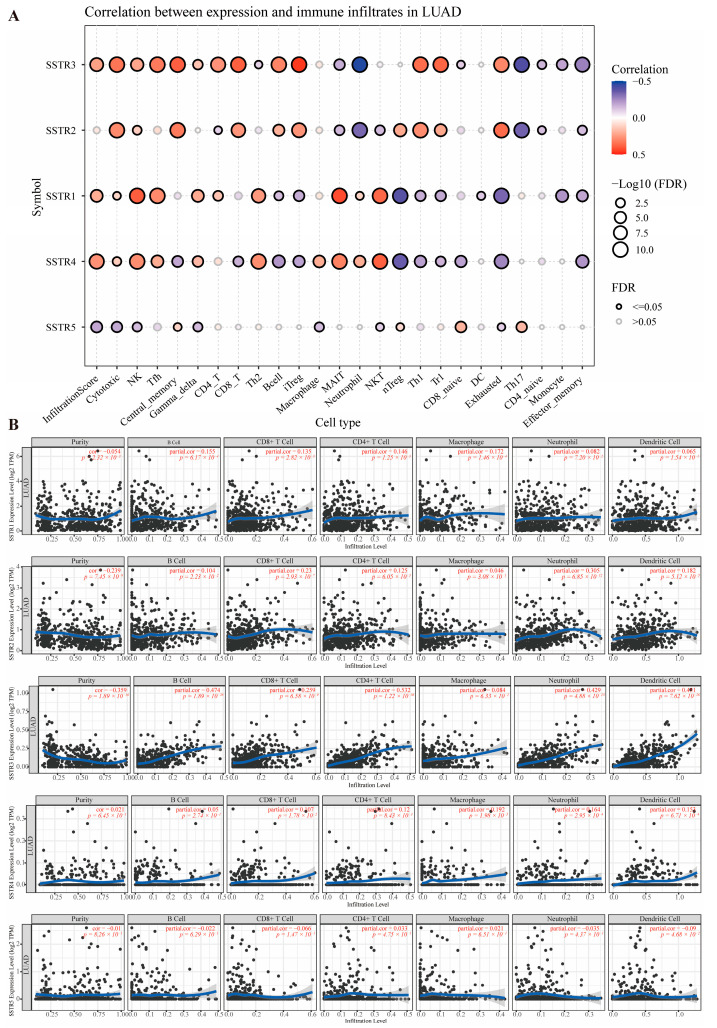
Correlation analysis between SSTR family expression and immune cell infiltration levels. (**A**) The size of the bubbles reflects the level of significance (expressed as −Log10 (FDR)), and the bubble color indicates the correlation coefficient (Correlation), ranging from blue (negative correlation) to red (positive correlation). Black hollow circles denote significant correlations (FDR ≤ 0.05), while gray hollow circles indicate non-significant correlations (FDR > 0.05). (**B**) Scatterplot showing the relationship between SSTR family expression and immune cell infiltration levels in the TIMER database. Each point represents a sample; the blue, smoothed curve indicates the trend line fitted by the local regression (LOESS) method, and the gray area represents the 95% confidence interval.

The study explored the correlation between different growth inhibitory receptor subtypes and various immune cell types. The SSTR family exhibits significant correlations with multiple immune cell types (Figure 3A). Overall, SSTR3 exhibited the strongest and most extensive significance. Its high expression showed a significant negative correlation with tumor purity (*p* = 1.89 × 10^−16^, partial correlation coefficient = −0.359) and a strong positive correlation with the vast majority of immune cells (particularly CD4+ T cells, *p* = 1.22 × 10^−36^, partial correlation coefficient = 0.532). SSTR1 showed significant correlations with immune cells (B cells, CD8+, CD4+ T cells) and macrophages (*p* < 0.05). SSTR2 exhibited an extremely strong positive correlation with neutrophil infiltration (*p* = 6.85 × 10^−12^, partial corr = 0.305). SSTR5 correlations were generally weak, with only dendritic cells showing a marginally significant association. SSTR4 expression was independent of tumor purity but significantly positively correlated with multiple immune cell infiltrates, including macrophages (*p* = 1.98 × 10^−5^, partial corr = 0.192), neutrophils (*p* = 2.95 × 10^−4^, partial corr = 0.164), and dendritic cells (*p* = 6.71 × 10^−4^, partial correlation = 0.153). Meanwhile, SSTR4 showed significant positive correlations with CD4+ T cells (*p* = 8.43 × 10^−3^, partial correlation = 0.120) and CD8+ T cells (*p* = 1.78 × 10^−2^, partial correlation = 0.107) (Figure 3B).

### 2.4. Functional Enrichment and Protein Interaction Network Analysis

**Figure 4:**
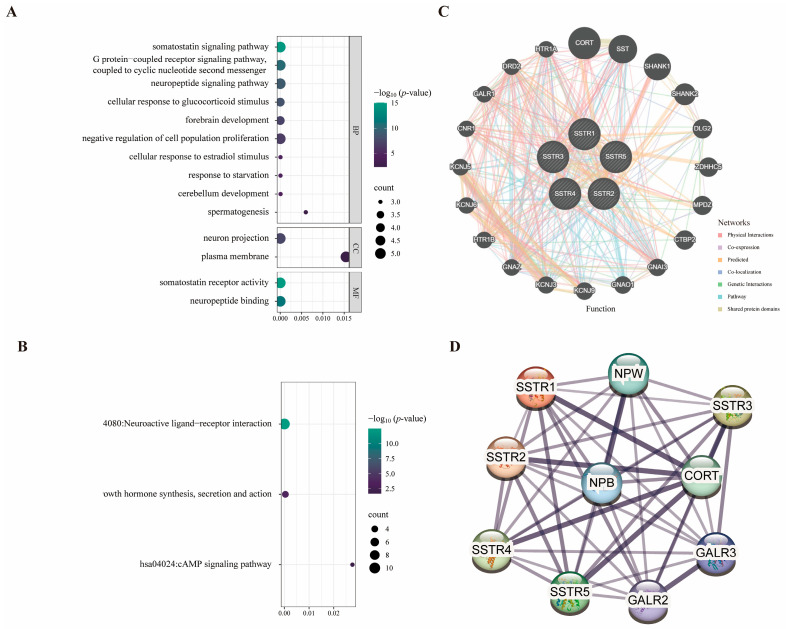
Protein interaction networks and SSTR family signaling pathways. (**A**,**B**) Plot of the results of gene enrichment analysis, demonstrating the enrichment of genes in different biological pathways and functions. The vertical axis is −log10 (*p*-value), which indicates the enrichment significance; the horizontal axis is the correlation statistical value; the color of the dots corresponds to the −log10 (*p*-value) range, and the size of the dots represents the number of enriched genes (COUNT). (**C**) The GeneMANIA functional association network analysis diagram combines several interaction networks, including physical interactions, co-expression, predicted interactions, colocalization, genetic interactions, pathway associations, and shared protein domains, in order to predict gene functions and their associated relationships. (**D**) Protein–protein interaction network diagram constructed based on the STRING database: Nodes represent proteins, and edges denote interaction relationships; thicker lines indicate higher confidence levels.

To elucidate the multidimensional roles of the SSTR family in LUAD, we integrated functional annotation, pathway, and network data, and predicted potential interactions using GeneMANIA (accessed on 12 April 2025). Significant enrichment signals are exhibited by the SSTR family across multiple levels, including biological processes, molecular functions, KEGG pathways, and protein interaction networks. The SSTR family demonstrated the highest significance in the “growth inhibitor signaling pathway” and “GPCR signaling pathway” among biological processes. In terms of molecular functions, “growth inhibitor receptor activity” and “neuropeptide binding” exhibited the highest significance, with “neuropeptide binding” standing out as the most significant (Figure 4A). In the KEGG analysis, the “Neuroactive ligand-receptor interactions” pathway was the most significant, involving the largest number of genes. The “Synthesis, Secretion, and Action of Growth Hormones” pathway was the second most important. Although the “cAMP signaling pathway” pathways involve a large number of genes, their enrichment scores were relatively low (Figure 4B). In addition, we constructed an integrated multi-interaction network to predict gene functional associations based on the GeneMANIA database (Figure 4C). The network incorporated multiple evidence types, with physical interactions constituting the predominant edge type (77.64%), followed by co-expression (8.01%), genetic interactions (2.87%), and shared protein domains. This framework enabled the identification of a core neuro-signaling module comprising SSTRs (SSTR1-5), potassium channel subunits (KCNJ3/5/6/9), G-protein components (GNA2/3/O1), and synaptic scaffolding proteins (DLG2/SHANK1), demonstrating how heterogeneous interaction data can elucidate coherent functional gene sets in neural signaling pathways. Based on the STRING database, a protein–protein interaction (PPI) network was constructed to elucidate the interactions among the SSTR family members (Figure 4D).

### 2.5. Tangeretin Inhibits Lung Cancer Cell Proliferation, Migration, and Invasion by Regulating Somatostatin Receptor 4

**Figure 5:**
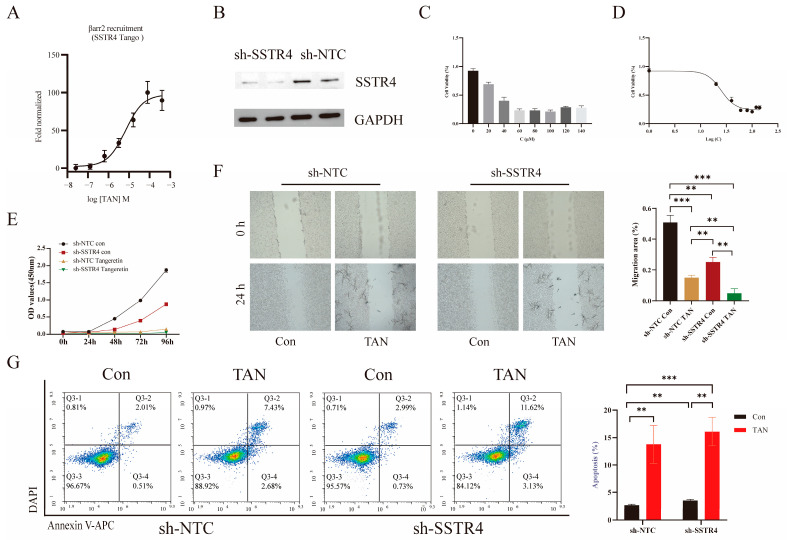
Tangeretin (TAN) inhibits the proliferation, migration, and invasion of lung cancer cells. (**A**) Cells were stimulated with TAN at the following concentrations: 400 μM, 80 μM, 16 μM, 3.2 μM, 0.64 μM, 0.128 μM, and 0.0256 μM. (**B**) Western blot of SSTR4 knockdown in A549 cells. sh-NTC, non-targeting control shRNA; sh-SSTR4, SSTR4-specific shRNA. (**C**) Cell proliferation capacity was assessed using the CCK-8 assay. A549 cells that had been stably transfected with sh-NTC and sh-SSTR4 were treated with TAN at concentrations of 0, 20, 40, 60, 80, 100, 120, or 140 μM for 72 h. The optical density (OD) was then measured at 450 nm. (**D**) Cellular viability was assessed by CCK-8 assay across a concentration–response series (mean ± SD, *n* = 3). Data were fitted to a four-parameter variable-slope model (log_10_ [concentration] versus normalized response), returning an IC_50_ of 24.7 μM (95% confidence interval: 22.1–27.8 μM). (**E**) Stably transfected sh-NTC and sh-SSTR4 cells were treated with 100 μM TAN and analysed at 0, 24, 48, 72, and 96 h post-treatment. (**F**) Representative images of wound healing assays were captured under a microscope. Images were acquired at 0 and 24 h post-scratch in sh-NTC and sh-SSTR4 cells. All *p*-values were determined using a two-tailed unpaired Student’s *t*-test (** *p* < 0.01, *** *p* < 0.001). (**G**) Flow cytometry using Annexin V-APC/DAPI double staining analysed apoptosis. After DAPI gating to exclude dead cells, the scatter plot represented Annexin V-APC fluorescence on the axis of abscissas and DAPI fluorescence on the axis of ordinates. The lower right quadrant (Q3-4) indicated early apoptosis, and the upper right quadrant (Q3-2) indicated late apoptosis.

Based on our group’s preliminary, unpublished work, it appears that TAN may interact with SSTR4. However, the biological function and downstream signaling mechanisms of this interaction remain unclear. To investigate whether TAN functions via SSTR4, we first conducted PRESTO-Tango experiments, which revealed that TAN induces β-arrestin recruitment in a concentration-dependent manner (Figure 5A). Building on this, we established a non-targeted control (sh-NTC) cell model and a stable SSTR4 knockdown (sh-SSTR4) cell model in the A549 cell line. Western blot analysis confirmed efficient and stable SSTR4 knockdown (Figure 5B).

We evaluated the anticancer potential of TAN against A549 cells via the CCK-8 assay. Results demonstrated that TAN effectively reduced A549 cell viability in a concentration-dependent manner, significantly inhibiting cell proliferation with an IC_50_ value of 24.7 μM (Figure 5C,D). Based on this finding, we selected 24.7 μM TAN as the optimal concentration for subsequent experiments. Functional assays revealed that either SSTR4 knockdown or TAN treatment alone effectively reduced cell viability in A549 cells (Figure 5E). Notably, the combination of both interventions produced a marked enhancement.

Scratch assay results confirmed that TAN significantly inhibited A549 cell migration, with enhancements observed when combined with SSTR4 knockdown. Intergroup comparisons revealed significant differences among the SSTR4 knockdown, TAN treatment, and combined treatment groups (Figure 5F). Furthermore, Annexin V-APC/DAPI double-stained flow cytometry revealed that both SSTR4 knockdown and TAN treatment independently induced significant apoptosis in A549 cells. Crucially, the total apoptosis rate (early + late, Q2 + Q4 quadrants) induced in the sh-SSTR4 TAN group was significantly elevated by 12.23% compared to the sh-NTC control group (Figure 5G).

### 2.6. Transcriptome Analysis Identifies Key Genes Involved in Synergistic Interactions

**Figure 6:**
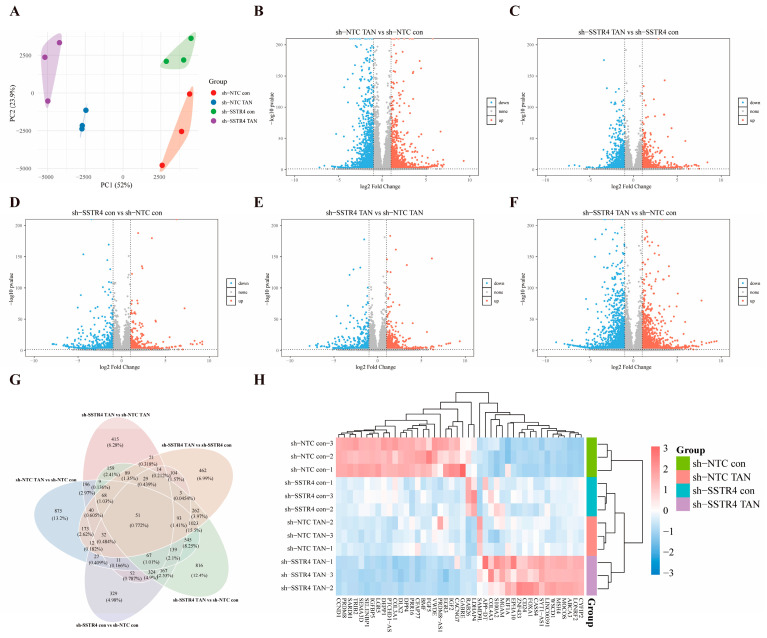
Transcriptomic analysis reveals molecular features of SSTR4 knockdown synergizing with TAN. (**A**) PCA analysis shows the overall distribution of gene expression profiles in A549 cells under different treatment conditions. Each dot = one biological replicate; colours: sh-NTC con (red), sh-NTC TAN (blue), sh-SSTR4 con (green), sh-SSTR4 TAN (purple). (*n* = 3 independent cultures per group) (**B**–**F**) Volcano plots. Red/blue: significantly up/downregulated genes. Criteria: (two-sided Wald test, FDR < 0.05 (Benjamini–Hochberg), |log_2_FC| ≥ 1. Gray: non-significant). (**G**,**H**) Heatmap of 51 common genes. Row-wise z-score calculated from DESeq2 variance-stabilizing-transformed counts. Columns represent individual RNA-seq samples (*n* = 3 biologically independent replicates per group). Colour scale: red = high, blue = low expression. Gene clustering: Euclidean distance + Ward linkage. Differential-expression criteria as in (**B**–**F**).

To elucidate the molecular mechanism underlying the synergistic suppression of malignant phenotypes in A549 cells by SSTR4 knockdown and TAN, we conducted an RNA-seq analysis on the sh-NTC, sh-NTC TAN, sh-SSTR4, and sh-SSTR4 TAN experimental groups based on prior pharmacological findings. This aimed to reveal the underlying differential transcriptional regulatory networks. Results showed that the first two principal components (PC1 and PC2) collectively accounted for 75.9% of the variance, effectively revealing the primary effects of experimental interventions. Specifically, the separation trend of PC1 (52%) was primarily attributable to the treatment effect of TAN, while PC2 (23.9%) significantly distinguished the genotype differences between the SSTR4 knockdown (sh-SSTR4) and control (sh-NTC) groups (Figure 6A). This analysis provided crucial grouping criteria for subsequent screening of differentially expressed genes.

To investigate the molecular mechanism by which SSTR4 knockdown enhances TAN’s anticancer activity, we performed RNA-seq analysis on A549 cells from different treatment groups and visualized the distribution of differentially expressed genes using a volcano plot. Differential analysis revealed that in the control background (sh-NTC), TAN treatment (sh-NTC TAN vs. sh-NTC con) induced significant changes in 3362 genes (|log_2_FC| ≥ 1, FDR < 0.05), comprising 1713 upregulated and 1649 downregulated genes (Figure 6B). In the SSTR4 knockdown background (sh-SSTR4), TAN treatment (sh-SSTR4 TAN vs. sh-SSTR4 con) reduced the number of affected genes to 2477 (1358 upregulated, 1092 downregulated) (Figure 6C). Notably, SSTR4 knockdown alone (sh-SSTR4 con vs. sh-NTC con) induced expression changes in 1465 genes (788 upregulated, 677 downregulated) (Figure 6D), sh-SSTR4 TAN vs. sh-NTC TAN upregulated 912 genes and downregulated 666 genes (Figure 6E). Most critically, the combined treatment group exhibited the most extensive transcriptional reprogramming compared to the blank control group (sh-SSTR4 TAN vs. sh-NTC con), identifying 3845 differentially regulated genes (2147 upregulated, 1698 downregulated) (Figure 6F).

After completing differential gene screening across comparison groups, we visualized the five sets of differentially expressed genes using Venn diagrams to further analyze the specificity and overlap of transcriptional regulation under different treatment conditions. The results revealed a common intersection comprising 51 genes (Figure 6G). These genes exhibited significant changes across all treatment conditions and may represent a core transcriptional regulatory network mediating the enhancements of SSTR4 knockdown and TAN.

For a visual representation of the expression patterns of the 51 core genes across the experimental groups, an expression heatmap was generated with the bioinformatics platform. The heatmap analysis clearly reveals the unique gene expression profiles of each group. Compared to the sh-NTC control group, it specifically upregulated genes such as CCND1, PRDM8, and SARDH, while downregulating genes including CYFIP2, LONRF2, and ABCA3. In contrast, sh-NTC TAN showed no significant changes in gene expression except for a marked upregulation of SAMD9L. In the sh-SSTR4 con group, only LDHAP4 and RAB26 showed upregulation overlapping with the sh-NTC control group. Crucially, the gene expression profile of the sh-SSTR4 knockdown combined with TAN treatment group exhibited a nearly completely opposite pattern to that of the sh-NTC control group (Figure 6H). The results above indicate that SSTR4 knockdown exhibits a significant enhancement with TAN.

### 2.7. GO and KEGG Enrichment Analysis of Differentially Expressed Genes

To investigate the primary biological functions of the differentially expressed genes, GO and KEGG enrichment analyses were performed. The corresponding results, including the top 10 significantly enriched GO terms and KEGG pathways and their respective *p*-values and FDR, can be found in Supplementary Figure S1.

### 2.8. Identification and Experimental Validation of Hub Genes Based on PPI Network Analysis

**Figure 7:**
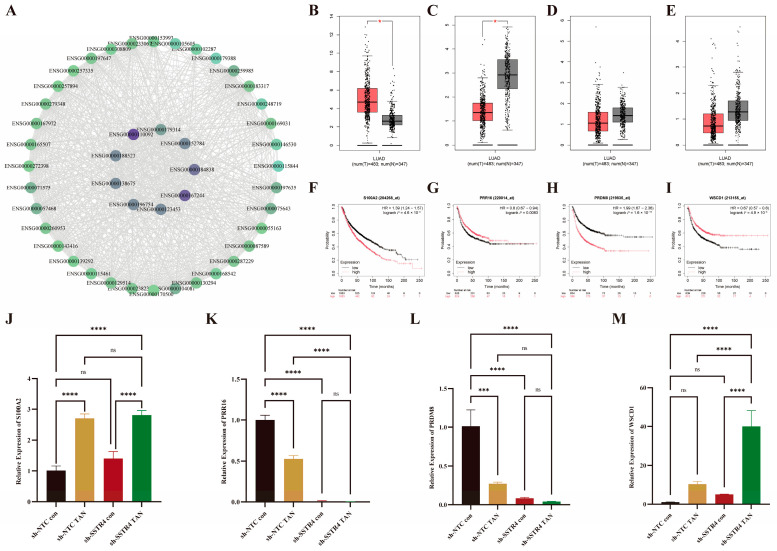
Core Gene PPI Network and Clinical Validation. (**A**) Core nodes generated using the Cytoscape-MCC algorithm based on STRING data; node size and purple-green gradient reflect degree values, with gray lines indicating protein interactions. (**B**–**E**) Expression comparison of Cytoscape -selected core genes (count ≥40) using the GEPIA2 platform. Box plots indicate * *p* < 0.05. (**F**–**I**) Kaplan–Meier survival curves comparing survival probabilities between the high-expression group (red) and low-expression group (black). The vertical axis represents survival probability, and the horizontal axis represents follow-up time. (**J**–**M**) RT-PCR quantification of SSTR4 transcript levels in A549 cells. Values were normalized to GAPDH and expressed as fold-change relative to sh-NTC control (assigned = 1). Data represent mean ± SD from three independent biological experiments, each performed in technical triplicate. Statistical significance was evaluated by one-way ANOVA followed by Tukey’s multiple-comparison test: ns, not significant (*p* ≥ 0.05), *** *p* < 0.001, **** *p* < 0.0001.

A total of 51 core genes were identified through Veen intersection analysis. Cytoscape_v3.10.3 association analysis revealed that 9 of these genes (count ≥ 40) were highly connected hub genes (Figure 7A). Further clinical correlation evaluation of these 9 genes demonstrated that *S100A2* was significantly upregulated in LUAD tissues, while *PRR16*, *PRDM8*, and *WSCD1* were significantly downregulated (Figure 7B–E). All three were associated with poor overall survival (log-rank test *p* < 0.05). Low expression of *S100A2* and *PRDM8* was correlated with better survival, whereas high expression of *PRR16* and *WSCD1* was associated with improved survival (Figure 7F–I). Subsequently, RT-PCR validated the expression of four core genes identified in the transcriptome analysis. Following TAN treatment, mRNA levels of *S100A2* and *WSCD1* were significantly elevated, while those of *PRR16* and *PRDM8* were markedly reduced (Figure 7J–M), consistent with sequencing results.

## 3. Discussion

This study systematically reveals the novel role of SSTR4, a member of the SSTR family, as a potential therapeutic target for LUAD through an integrated approach combining bioinformatics analysis and cellular model experiments. Our findings expand the current understanding of the functional diversity of the SSTR family in lung cancer and validate TAN as a potential SSTR4 ligand. This TAN-SSTR4 signaling axis mediates antitumor effects, providing novel insights and experimental evidence for targeted therapy in LUAD.

Bioinformatic analysis revealed that SSTR4 expression is significantly downregulated in LUAD tissues, and its low abundance is associated with advanced TNM stage and shorter overall survival, indicating that SSTR4 may serve as an independent prognostic protector. During tumor progression, promoter methylation of SSTR4 increases markedly, whereas its mRNA level is positively correlated with the infiltration of CD4^+^ T cells, M1 macrophages, and other immune subpopulations, supporting the notion that epigenetic silencing of SSTR4 fosters an immunosuppressive micro-environment and accelerates tumor advancement [35,36].

At a functional level, we revealed the link between the natural compound TAN and SSTR4 in regulating the malignant behavior of A549 cells. Experimental results showed that targeting SSTR4 significantly increased A549 cells’ sensitivity to TAN. The two agents exhibited marked enhancements in inhibiting cell proliferation and migration whilst inducing apoptosis. This indicates that the functional state of SSTR4 is a key determinant of the response of A549 cells to TAN. Further studies showed that TAN effectively suppresses A549 cell proliferation in a concentration-dependent manner. This finding is consistent with previous reports on other cancer cell types and supports the potential of TAN as a broad-spectrum antitumor agent [16,37]. These antitumor phenotypes, corroborated by bioinformatics findings on SSTR4, strongly support the hypothesis that SSTR4 plays a crucial role in LUAD.

To thoroughly dissect its mechanism of action, we further employed transcriptomic sequencing as an unbiased research strategy. By systematically comparing RNA-Seq data between the sh-NTC control group and the sh-SSTR4 knockdown group following TAN treatment, we discovered that SSTR4 acts as a key negative regulator, limiting TAN-induced excessive ECM activation and abnormal signal transduction. The absence of SSTR4 shifts TAN’s primary effect from ECM-cytoskeletal regulation to interference with core cellular functions, including calcium homeostasis disruption, energy metabolism disorder, and dysregulation of “cancer pathways.” This is accompanied by transient activation of oncogenic signals at the genomic level, ultimately manifesting as a significant anticancer effect. This “an initial surge in pro-survival signals followed by rapid cytotoxicity” transcriptional pattern suggests that SSTR4 knockdown releases not oncogenic potential but suppressed TAN cytotoxic effects; the substantial upregulation of oncogenic signals can be interpreted as the genomic expression of cellular stress responses preceding cell death [38]. Notably, the core transcriptional programs of sh-NTC control cells and sh-SSTR4 TAN-treated cells diverged markedly, indicating that the pharmacological activity of TAN is predominantly channeled through SSTR4. In its absence, off-target engagement or compensatory pathway rewiring may result in an unexpected reconfiguration of the transcriptome. This phenomenon is similar to the ”differential signal rewiring” that causes altered drug sensitivity in STK11-mutant lung cancer models [39].

In summary, this study confirms that the natural compound TAN significantly inhibits LUAD cell proliferation and migration by activating SSTR4. It clarifies the key tumor-suppressing function of SSTR4 in LUAD and its importance as a potential therapeutic target, while also revealing the preliminary molecular mechanisms by which TAN regulates SSTR4 downstream signaling pathways. This work establishes a foundation for evaluating prognosis and treatment response by measuring SSTR4 expression levels in LUAD patients while also offering new insights into the development of SSTR4-targeted therapeutic strategies for patients with advanced LUAD.

Future research should validate the universality of SSTR4 regulation across multiple cell lines, including PC-9 and H1299, and integrate in vivo models with multi-omics time-series data to systematically characterize its dynamic alterations within the tumor microenvironment. Concurrently, the interaction network between SSTR4 and established lung cancer driver genes should be elucidated, and the differential efficacy of TAN across molecular subtypes of LUAD should be evaluated. Additionally, dedicated synergy experiments will be conducted to quantify the in vitro and in vivo synergistic potency of SSTR4 and TAN. This will provide a more comprehensive molecular regulatory map and advance SSTR4-guided precision treatment strategies toward clinical application.

## 4. Materials and Methods

### 4.1. Expression Analysis of SSTR Family in LUAD

Using the Genomic Somatic Cancer Atlas (GSCA)—an integrative genomics and immunogenomics web portal (https://guolab.wchscu.cn/GSCA/#/ (accessed on 28 March 2025))—we conducted a systematic pan-cancer analysis of SSTR family gene expression across 10,471 samples spanning 33 cancer types. The LUAD subset comprised 576 specimens: 517 tumor tissues, 59 normal tissues, and 58 matched pairs, enabling differential expression profiling between LUAD and normal lung tissue. To validate stage-specific alterations of SSTR family expression during LUAD progression, we retrieved stage-stratified expression data from The Cancer Genome Atlas (TCGA) via the University of Alabama at Birmingham Cancer Data Analysis Portal (UALCAN, https://ualcan.path.uab.edu/ (accessed on 28 March 2025)), thereby assessing the potential roles of the SSTR family in LUAD initiation and development.

### 4.2. SSTR Prognostic Signatures in LUAD

Expression profiles of the SSTR gene family in LUAD were obtained from the TCGA module on the UALCAN portal (https://ualcan.path.uab.edu/ (accessed on 28 March 2025)). Survival associations were analyzed within the “lung cancer” module of the Kaplan–Meier Plotter public platform (https://www.kmplot.com/analysis/ (accessed on 28 March 2025)). The platform employs the JetSet best probe set as the default gene proxy; after array-quality control, 2166 patients were retained (2850 biased arrays excluded). Overall survival (OS) was estimated using the Cox proportional-hazards model, with statistical significance set at *p* < 0.05.

### 4.3. Immune Contexture Profiling by SSTR Expression in LUAD

In this study, we integrated information from the GSCA and TIMER (https://compbio.cn/ (accessed on 7 April 2025), *n* = 515) databases to conduct an in-depth analysis of LUAD samples. We obtained expression data for growth suppression receptors (SSTR1-5) and associated immune cell infiltration information from the GSCA database, and explored the correlation between SSTR family subtypes and immune cell infiltration levels through correlation analysis.

### 4.4. Functional and Interaction Analyses of SSTR Family in LUAD

This study employed the DAVID database (https://davidbioinformatics.nih.gov (accessed on 12 April 2025)) to conduct Gene Ontology (GO) functional annotation and Kyoto Encyclopedia of Genes and Genomes (KEGG) pathway enrichment analysis on the target gene set. Data analysis and visualization were performed using the online bioinformatics platform (https://www.bioinformatics.com.cn/ (accessed on 12 April 2025)). Furthermore, by integrating multiple association evidence—including protein–protein interactions, co-expression, and pathway information—a functional prediction network was constructed using GeneMANIA database (version 3.6.0; https://genemania.org/ (accessed on 12 April 2025)) and the STRING database (https://cn.string-db.org/ (accessed on 12 April 2025)). This network systematically elucidates the key biological processes involving SSTR gene family members and their molecular interaction mechanisms, thereby revealing potential functional associations and regulatory networks within this gene family.

### 4.5. Cell Culture and Conditions

The human LUAD cell line A549 was procured from the Type Culture Collection (ATCC). The cells were cultivated in RPMI 1640 medium (Gibco, Thermo Fisher Scientific, Waltham, MA, USA) with the addition of 10% fetal bovine serum (FBS, Vivacell, Denzlingen, Germany) and 1% Penicillin-Streptomycin (Gibco, Thermo Fisher Scientific, Waltham, MA, USA). The cells were cultivated in a constant-temperature incubator maintained at 37 °C, with 5% CO_2_ and saturated humidity. Unless otherwise indicated, all cells utilized in this experiment were cultivated under the aforementioned conditions.

### 4.6. Generation of Somatostatin Receptor 4-Knockdown Stable Cell Lines

Linearize pLKO.1-TRC using EcoRⅠ + AgeⅠ double digestion, ligate with annealed sh-SSTR4 fragments and their control sequences, then transform and sequence for verification; Co-transfected with psPAX2/pMD2.G into 293T cells for viral packaging. Viral particles were harvested from cell supernatants after 48 h by centrifugation. Infected A549 cells were cultured under continuous selection with 1.5 mg/mL puromycin for one week. Stable knockdown was validated by Western blot analysis.

### 4.7. Western Blotting

Total protein was extracted from stable cell lines. After quantification using the Thermo Scientific Pierce BCA Protein Quantification Kit, proteins were separated by SDS-PAGE gel electrophoresis and subsequently transferred to polyvinylidene fluoride (PVDF) membranes (Omni, Shanghai, China). The membrane was blocked with 5% skim milk powder for 30 min, followed by overnight incubation at 4 °C with the following primary antibodies: anti-SSTR4 (ImmunoWay, Plano, TX, USA, diluted 1:1000) and anti-GAPDH (Proteintech, Rosemont, IL, USA, diluted 1:10,000). The membrane was then incubated with the secondary antibody at room temperature for 1 h. Finally, visualization was achieved using ECL chemiluminescent reagent (Omni, Shanghai, China).

### 4.8. Parallel Receptor-Ome Expression and Screening via Transcriptional Output (PRESTO)—Tango Assay

When HEK-293T cells in 6 cm culture dishes reached 80% confluence, they were transfected with SSTR4-Tango, β-arrestin2-TEV, and TRE-Tight-Luc plasmids at a 2:1:1 ratio. 24 h post-transfection, cells were seeded at 3 × 10^4^ cells/well into 96-well plates and cultured overnight. Subsequently, replace the medium with fresh medium containing TAN at final concentrations ranging from 400 μM to 0.00256 μM. After 18 h of incubation, add 50 μL of Bright-Glo substrate (Promega, Madison, WI, USA, #E2610). Mix thoroughly and immediately measure the fluorescence intensity.

### 4.9. Cell Counting Kit-8 (CCK-8) Assay

Cell proliferation was measured using cell counting kit-8 (CCK-8) (Targetmol, Boston, MA, USA). Stably transfected sh-NTC and sh-SSTR4 cells (2 × 10^3^ cells per well) were seeded into 96-well plates (NEST). After 24 h, the cells were treated with TAN at respective concentrations. At the designated time point, prior to terminating the culture, 10 μL of CCK-8 reagent was added to each well. Then, the cells were incubated at 37 °C for 2 h. At the indicated time points, the absorbance at 450 nm was measured to determine the cell viability using the microplate reader (spectramax id3, Molecular Devices, San Jose, CA, USA). The data are representative of three independent experiments in triplicate.

### 4.10. Cell Apoptosis Analysis

Apoptosis detection was performed using flow cytometry. Cells were seeded into six-well plates at a density of 7 × 10^4^ cells per well. After treatment, the cells were collected by centrifugation at 500 rpm for 5 min and washed twice with 1× PBS. The cells were resuspended in 125 μL of 1× binding buffer supplemented with 1.25 μL each of APC-labeled Annexin V and DAPI. The mixture was incubated in the dark for 15 min. Finally, analysis was performed on a NovoCyto flow cytometer (NovoCyto, Beijing, China).

### 4.11. Wound Healing Assay

Cells were seeded at a density of 6 × 10^5^ cells per well in 12-well plates that contained pre-sterilized scratch inserts (BMS). The inserts were gently pressed to expel air bubbles. Once the cells reached 90% confluence, the inserts were carefully removed to create uniform scratch wounds. The cells were washed with PBS, then transferred to a medium supplemented with 10% FBS. Add pre-labeled concentrations of TAN or DMSO (as a control) to each well. Capture scratch images at 0 h and 24 h using an inverted microscope (Olympus IX73, Tokyo, Japan) under fixed field-of-view conditions.

### 4.12. RNA Extraction and RNA Sequencing

Total RNA was isolated from drug-treated and control cells of both sh-NTC and sh-SSTR4 groups using the Eastep^®^ Super Total RNA Extraction Kit. Samples were sent to Benagen Technology (Wuhan, Hubei, China) for library preparation and transcriptome sequencing. Following quality control of the raw sequencing data, differential expression analysis was performed using the DESeq2 package in R to identify differentially expressed genes (DEGs) between the two groups. Subsequently, GO functional annotation and KEGG pathway enrichment analyses were conducted on the identified DEGs based on the DAVID database via the Majorbio Cloud Platform, with FDR < 0.05 set as the threshold for statistical significance.

### 4.13. cDNA Synthesis and Reverse Transcription Quantitative Polymerase Chain Reaction (RT-PCR)

Total cellular RNA was extracted using TRIzol reagent (Takara, Kusatsu, Shiga, Japan). cDNA was synthesized from 400 ng of total RNA using the PrimeScript™ RT Master Mix (Takara, Dalian, Liaoning, China) in a 20 μL reaction volume. RT-PCR analysis was performed on a QuantStudio™ 5 system (Thermo Fisher Scientific, Waltham, MA, USA) using TB Green Premix Ex Taq™ II (Takara, Dalian, Liaoning, China) with diluted cDNA as the template. The thermal cycling protocol was as follows: initial denaturation at 95 °C for 30 s, followed by 40 cycles of 95 °C for 5 s and 60 °C for 10 s. GAPDH was used as an endogenous reference gene for normalization (Table 1). Relative expression levels of circular RNA were calculated using the 2^–ΔΔCt^ method.

## 5. Conclusions

This study uses bioinformatics techniques to confirm that low SSTR4 expression is an independent predictor of advanced staging and poor prognosis in LUAD. In vitro experiments demonstrate that SSTR4 knockdown enhances the inhibitory effect of TAN on A549 cell proliferation and migration. This suggests that SSTR4 has dual value as both a prognostic marker and a potential regulatory node. However, these findings are currently limited to phenotypic observations of a single cell line and lack validation in spontaneous models. The long-term toxicity and specific molecular mechanisms of TAN remain to be explored. Future work will involve expanding clinical sample sizes, replicating phenotypes across multiple cell lines and animal models, and conducting preclinical pharmacokinetic/toxicokinetic studies. The aim is to provide intervention strategies for patients with low SSTR4 expression.

## Figures and Tables

**Table 1:** Primers used for RT-PCR.

Gene	Forward (5′–3′)	Reverse (5′–3′)
GAPDH	GGAGCGAGATCCCTCCAAAAT	GGCTGTTGTCATACTTCTCATGG
S100A2	CCAAGAGGGCGACAAGTTCAAG	CTGCTGGTCACTGTTCTCATCC
PRR16	AGCTGGAGGATGAGATGACTGAC	TCTAGGCTGGAGGCTGTTGTG
PRDM8	GCCATCCACAGACTTCCACAAC	CCGCTACCGCTGCTGAGG
WSCD1	CGCCTCGTGGTGGTCCTC	CCGCTCCTCGCTCACAGAC

## Data Availability

The RNA-seq data generated in this study have been deposited in the NCBI BioProject database under accession number PRJNA1358239. All other data supporting the findings of this article are publicly available from the following online databases: The pan-cancer analysis and immune correlation were performed using the GSCA platform (https://guolab.wchscu.cn/GSCA/#/ (accessed on 28 March 2025)). The validation of gene expression across clinical stages was conducted via the UALCAN portal (https://ualcan.path.uab.edu/ (accessed on 28 March 2025)). Survival analysis was performed using the Kaplan–Meier Plotter (https://kmplot.com/analysis/ (accessed on 28 March 2025)). Functional enrichment analysis was carried out with the DAVID database (https://davidbioinformatics.nih.gov (accessed on 12 April 2025)). Protein–protein interaction networks were constructed using GeneMANIA (https://genemania.org (accessed on 12 April 2025)) and STRING (https://string-db.org/ (accessed on 12 April 2025)).

## Supplementary Materials

The following supporting information can be downloaded at https://www.mdpi.com/article/10.3390/ijms27021074/s1.

## Abbreviations

The following abbreviations are used in this manuscript:

LUAD	Lung adenocarcinoma
SSTR	somatostatin receptor
TAN	Tangeretin
GPCR	G protein-coupled receptor
GO	Gene Ontology
KEGG	Kyoto Encyclopedia of Genes and Genomes
