# Supplementary material for: Tangeretin Suppresses LUAD via SSTR4 Downregulation: Integrated Bioinformatics and Functional Validation
Source: Int J Mol Sci. 2026 Jan 21;27(2):1074. doi: 10.3390/ijms27021074 (PMC12842074; doi:10.3390/ijms27021074)
Supplement: Supplementary file 1 [file ijms-27-01074-s001.zip › ijms-4091390-supplementary.pdf]

Supplementary Data

Supplementary Figure S1. GO and KEGG enrichment analysis of differentially expressed genes

Through five sets of comparative GO and KEGG enrichment analyses, we systematically evaluated the impact of SSTR4 knockdown on TAN function. In the comparisons of the drug-treated groups (Fig. S1A, B), TAN treatment significantly activated pathways related to the extracellular matrix and plasma membrane in both the sh-NTC and SSTR4 knockdown backgrounds. The plasma membrane pathway exhibited the most significant gene enrichment under both conditions, and the extracellular region along with related structural components also showed high significance. In the knockdown background, drug treatment further induced extensive activation of pathways such as extracellular matrix organization, cell adhesion, and basement membrane. Although these pathways involved a limited number of genes, they remained highly significant. In the analysis of the knockdown groups (Fig. S1C, D), the absence of SSTR4 affected cellular signal transduction by remodeling the structure of the cell membrane and ion homeostasis, a process that displayed significant condition dependency. In the comparison of sh-SSTR4 con vs sh-NTC con, the effects were concentrated on the basement membrane pathway and calcium ion binding function. In contrast, the comparison of sh-SSTR4 TAN vs sh-NTC TAN shifted to significantly affecting the plasma membrane pathway. However, calcium ion binding function was disrupted across different conditions. To reveal the specific regulatory patterns of TAN treatment in the context of SSTR4 deletion, we performed a comparative analysis between "sh-SSTR4 TAN vs sh-NTC con" Among these, the plasma membrane pathway showed the strongest enrichment significance and involved the largest number of genes. Concurrently, significant gene enrichment was observed in pathways including cell adhesion, extracellular matrix, collagen-containing extracellular matrix, basement membrane, sequence-specific DNA binding, and extracellular matrix structural components. Among pathways related to the extracellular matrix, basement membrane, and their structural components, the number of enriched genes was relatively lower (Fig. S1E).

KEGG pathway analysis demonstrated that SSTR4 knockdown alters the signaling networks activated by TAN (Fig. S1F-J). Under normal SSTR4 expression, TAN primarily induced pathways related to ECM–receptor interaction, cytoskeleton remodeling, PI3K-Akt, and MAPK. Following SSTR4 knockdown, however, tangeretin-mediated signaling was markedly reprogrammed, characterized by disruption of basal calcium signaling and enrichment of pathways associated with cancer, along with specific post-translational modifications such as mucin-type O-glycan biosynthesis.

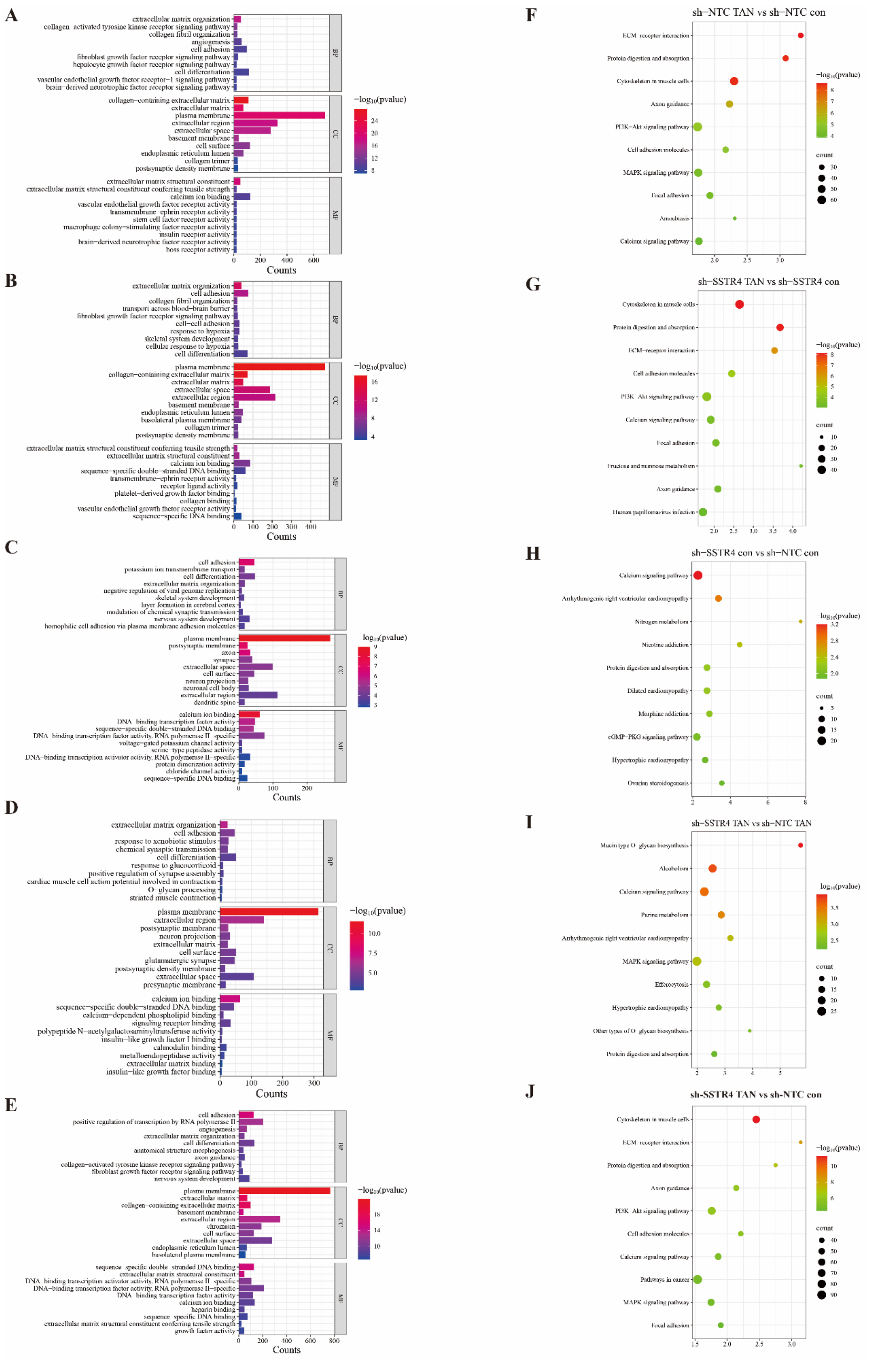

Figure S1. GO and KEGG enrichment analysis results for differentially expressed genes across five

experimental groups. A-E Top 10 most significantly enriched pathways across the three categories: Biological Process (BP), Cellular Component (CC), and Molecular Function (MF). Bar colors represent enrichment significance (-log10 (*p*-value)), with redder bars indicating stronger enrichment. Bar length reflects the number of genes enriched in that pathway, with longer bars denoting greater gene involvement in that function. A: sh-NTC TAN vs sh-NTC con. B: sh-SSTR4 TAN vs sh-SSTR4 con. C: sh-SSTR4 con vs sh-NTC con. D: sh-SSTR4 TAN vs sh-NTC TAN. E: sh-SSTR4 TAN vs sh-NTC con. F-J Top ten most significantly enriched pathways. Bubble color represents enrichment significance (-log10 (*p*-value)), with redder colors indicating more significant enrichment. Bubble size indicates the number of differentially expressed genes enriched in that pathway; larger bubbles involve more genes.
